# Acid-Sensing Ion Channel 1a in the amygdala is involved in pain and anxiety-related behaviours associated with arthritis

**DOI:** 10.1038/srep43617

**Published:** 2017-03-02

**Authors:** Youssef Aissouni, Abderrahim El Guerrab, Al Mahdy Hamieh, Jérémy Ferrier, Maryse Chalus, Diane Lemaire, Stéphanie Grégoire, Monique Etienne, Alain Eschalier, Denis Ardid, Eric Lingueglia, Fabien Marchand

**Affiliations:** 1 Clermont Université, Université d’Auvergne, Pharmacologie fondamentale et clinique de la douleur, F-63000 Clermont-Ferrand, France; 2 Inserm U1107 NEURO-DOL, F-63001 Clermont-Ferrand, France; 3 Centre Jean Perrin, ERTICA EA4677 Université d’Auvergne F-63001, Clermont-Ferrand, France; 4 CHU Clermont-Ferrand, F-63000 Clermont-Ferrand, France; 5 Université Côte d’Azur, CNRS, IPMC, Valbonne, France; 6 LabEx Ion Channel Science and Therapeutics, Valbonne, France

## Abstract

Chronic pain is associated with anxiety and depression episodes. The amygdala plays a key role in the relationship between emotional responses and chronic pain. Here, we investigated the role of Acid-Sensing Ion Channels 1a within the basolateral amygdala (BLA), in pain and associated anxiety in a rat model of monoarthritis (MoAr). Administration within the BLA of PcTx1 or mambalgin-1, two specific inhibitors of ASIC1a-containing channels significantly inhibited pain and anxiety-related behaviours in MoAr rats. The effect of PcTx1 was correlated with a reduction of c-Fos expression in the BLA. We examined the expression profile of ASICs and other genes in the amygdala in MoAr and sham animals, and found no variation of the expression of ASIC1a, which was confirmed at the protein level. However, an increase in the BLA of MoAr rats of both PI3Kinase mRNA and the phosphorylated form of Akt, along with Bdnf mRNA, suggest that the BDNF/PI3-kinase/Akt pathway might regulate ASIC1a in BLA neurons as demonstrated in spinal sensitisation phenomenon. We also observed changes in several kinase mRNAs expression (PICK1, Sgk1) that are potentially involved in ASIC1a regulation. These results show a crucial role of ASIC1a channels in the BLA in pain and anxiety-related behaviours during arthritis.

The affective and emotional component contributes to the unpleasantness and distress response of pain. It is usually manifested by anxiety, depression, stress, anger or fear episodes. These negative emotions arise particularly in a chronic pain context and are often dependent on pain intensity, duration and/or the perception by the individual^1^,^2^,^3^. The amygdala, a structure well known for its crucial role in emotion and affective disorders, has been more recently shown to participate in the relationship between chronic pain and emotional responses^4^,^5^,^6^,^7^,^8^,^9^. Among the different functionally distinct nuclei of the amygdala, the central nuclei (CeA) receives purely nociceptive information directly from the spino-parabrachial pathway and affect-related information from the lateral-basolateral nuclei network, and can modulate pain behaviour through activation of the brainstem descending pain control centres. On the other hand, the basolateral nucleus (BLA) is the major source of input to certain prefrontal cortical areas and could be critical for emotional/cognitive functions^4^,^5^,^8^,^9^. Electrophysiological and functional imaging studies in animals and humans show an increased response of the amygdala during a painful episode^5^,^7^. In contrast, pharmacological inhibition or lesion of the amygdala greatly reduces the emotional reactions related to pain without necessarily affecting the sensory responses [for review see ref. 5]. Finally, synaptic plasticity phenomena (*i.e.*, long term potentiation, sensitisation) taking place in the amygdala could partially explain the affective disorders associated with chronic pain^4^,^10^,^11^. However, the underlying mechanisms have only been partially elucidated.

Several studies have reported the involvement of ASIC1a channels, which form voltage-independent cation channels activated by extracellular acidification^12^, in peripheral and central (*i.e.*, spinal cord) sensitisation in different models of inflammatory, neuropathic and visceral pain^13^,^14^,^15^. In addition, ASIC1a is the most abundant ASIC channel expressed in the brain and is primarily responsible for acid-evoked currents in the amygdala^16^. ASIC2 subunits in the brain more likely behave as ASIC1a-modulatory subunits^17^. ASIC1a expressed in the amygdala has been implicated in aversive fear conditioning, anxiety, depression, and some learning and memory processes^16^,^17^,^18^,^19^. A contribution of post-synaptic ASIC currents to synaptic plasticity was recently demonstrated in the lateral amygdala^17^. However, the role of ASIC1a in the amygdala in relation with chronic pain and associated emotional disorders is still unknown. The aim of the present study was to investigate the role of ASIC1a channels in the BLA-dependent pain and anxiety-related behaviours associated with inflammatory pain in a rat model of arthritis^20^.

## Results

### Pharmacological inhibition of ASIC1a in the BLA reduced pain as well as anxiety related behaviours associated with arthritis

**Figure 1:**
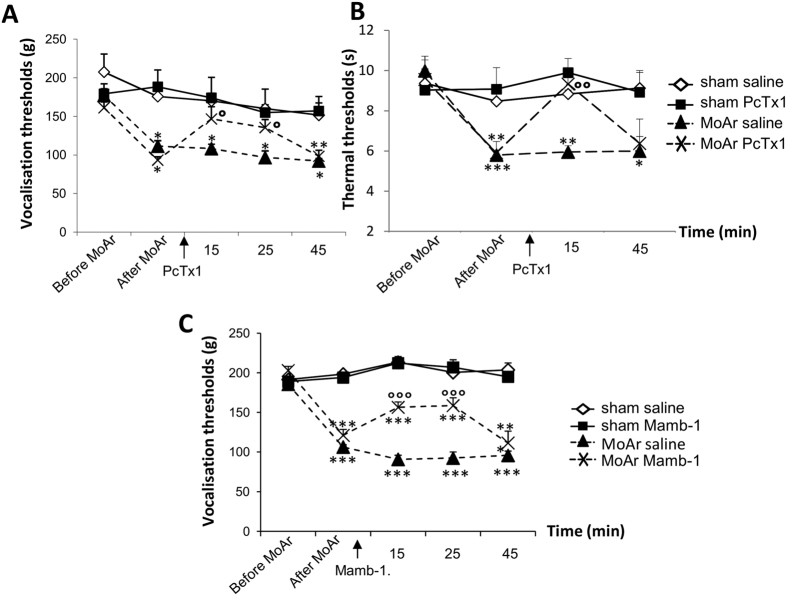
Effect of PcTx1 or mambalgin-1 (Mamb-1) bilateral microinjection (0.05 and 0.065 μg/rat/side, respectively, 15 min before the test) within the BLA of sham and MoAr rats on mechanical (**A** for PcTx1, **C** for manbalgin-1) and thermal (**B**, PcTx1 only) hyperalgesia using paw pressure and paw immersion test at 46 °C. Tests were performed 14–16 days following monoarthritis induction. Arrows indicated the injection of the inhibitor. n = 6–8 animals per group. Two-way repeated measures ANOVA followed by a Tuckey *post hoc* test. *p ≤ 0.05, **p ≤ 0.01, ***p ≤ 0.001 compared to sham saline group, °p ≤ 0.05, °°p ≤ 0.01, °°°p ≤ 0.001 compared to MoAr saline group.

To define the involvement of ASIC1a channels in mechanical and thermal hyperalgesia, the effect of intra-BLA administration of the ASIC1a-specific peptide inhibitor PcTx1^21^ in sham or MoAr rats was examined. As expected, intra-articular CFA administration significantly reduced vocalisation thresholds to mechanical pressure applied to the ankle at D14 compared to baseline (from 170 ± 5 g to 102 ± 4 g, Fig. 1A). Bilateral administration of PcTx1 within the BLA significantly relieved mechanical hyperalgesia in MoAr rats at 15 and 25 min compared to saline treated MoAr rats (140 ± 16 g and 131.5 ± 10 g, respectively, corresponding to a % of maximum possible effect (MPE) of 29.6 ± 10.6 and 23.7 ± 6.6%, respectively), while the administration of saline or PcTx1 within the BLA of sham rats did not change vocalisation thresholds (Fig. 1A).

Similar results were observed with thermal hyperalgesia. As shown in Fig. 1B, CFA administration significantly reduced thermal thresholds at D14 (from 9.8 ± 0.4 s to 5.8 ± 0.22 s). PcTx1 administration within the BLA significantly relieved thermal hyperalgesia in MoAr rats 15 min post administration compared to saline treated MoAr animals (9.3 ± 1.2 s corresponding to a % of MPE: 40 ± 12.6%), while bilateral administration of saline or PcTx1 within the BLA of sham rats did not change thermal thresholds. The effect of PcTx1 is therefore potent, especially on thermal hyperalgesia, but relatively short-lasting as thermal and vocalisation thresholds reached pre-administration value 45 min after PcTx1 injection (Fig. 1A and B).

To confirm the results obtained with PcTx1, the effect of intra-BLA administration of mambalgin-1, another specific peptide inhibitor of ASIC1a-containing channels^22^, in sham or MoAr rats was also evaluated. As observed in the previous experiment, intra-articular CFA administration significantly reduced vocalisation thresholds to mechanical pressure applied to the ankle at D14 compared to baseline (from 193 ± 4 g to 113 ± 4 g, Fig. 1C). Bilateral administration of mambalgin-1 within the BLA significantly relieved mechanical hyperalgesia in MoAr rats at 15 and 25 min compared to saline treated MoAr rats (156 ± 10 g and 159 ± 15 g, respectively, corresponding to a % of maximum possible effect (MPE) of 13.7 ± 4.2 and 14.6 ± 5.9%, respectively), while the administration of saline or mambalgin-1 within the BLA of sham rats did not change vocalisation thresholds (Fig. 1C).

**Figure 2:**
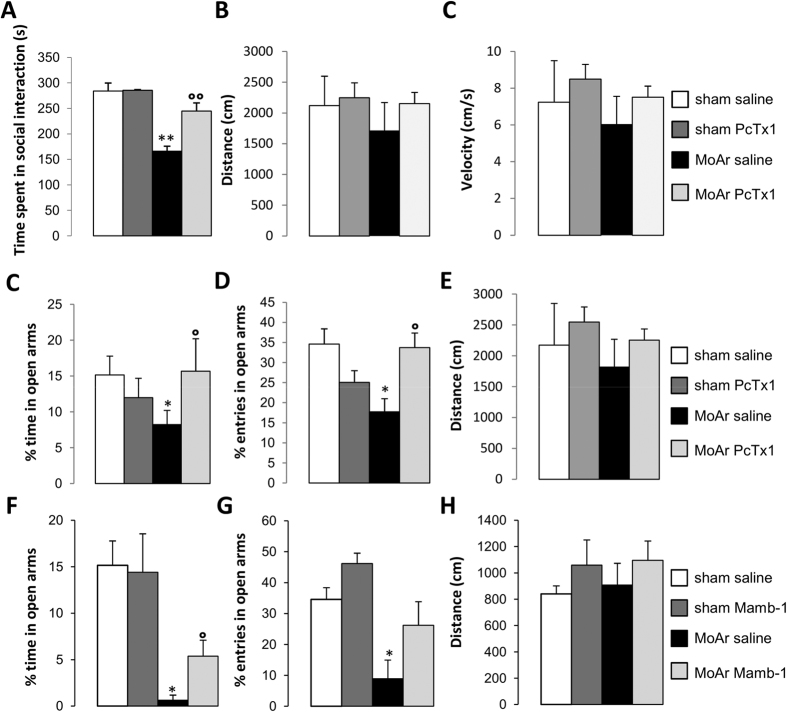
(**A**) Effect of PcTx1 bilateral microinjection (0.05 μg/rat/side, respectively, 15 min before the test) within the BLA of sham and MoAr rats on the time spent in social interactions. (**B**,**C**) Evaluation of locomotor activity in MoAr animals or sham rats injected or not with PcTx1 (**B**, distance travelled and **C**, velocity) during a 5 min session in the open field arena. (**C**–**H**) Effect of PcTx1 or mambalgin-1 bilateral microinjection (0.05 and 0.065 μg/rat/side, respectively, 15 min before the test) within the BLA of sham and MoAr rats on the the percentage of time spent in the open arms (**C** and **F**), the percentage of entries (**D** and **G**), and the distance travelled (**E** and **H**) in the elevated plus maze test after PcTx1 (**C–E**) or mambalgin-1 (**F–H**) injection. Tests were performed 14–16 days following monoarthritis induction. The movements of the animals in the EPM test were automatically recorded by a camera for 5 min and analyzed using Ethovision XT 8 (Noldus). n = 6–8 animals per group; One-way ANOVA followed by a Tuckey *post hoc* test. *p ≤ 0.05, **p ≤ 0.01 compared to sham saline group, °p ≤ 0.05, °°p ≤ 0.01 compared to MoAr saline group.

We further evaluated the effect of PcTx1 or mambalgin-1 on anxiety related behaviours. Regarding the social interaction test (Fig. 2A), a significant difference in the time spent in social interaction was observed between the different groups. In MoAr saline-treated animals, a significant decrease of the time spent in social interaction was observed compared to sham animals (Fig. 2A). This decrease was absent after bilateral administration of PcTx1 within the BLA of MoAr rats, while no effect of PcTx1 was detected in sham rats (Fig. 2A).

We assessed locomotor behavior 14 days following CFA injection and 15 min after PcTx1 or saline infusion within the BLA using the open field test. The distance and velocity were not statistically different between groups, excluding possible activity and/or motor impairment induced by CFA or ASIC1a inhibitor administration (Fig. 2B and C).

In the elevated plus maze test, we observed a significant decrease in the percentage of time and entries spent in the open arms in saline-treated MoAr rats compared to sham saline-treated (Fig. 2C,D,F and G), suggesting increased anxiety. Bilateral administration of PcTx1 (Fig. 2C and D) or mambalgin-1 (Fig. 2F and G) reversed (partially for mambalgin-1) anxiety related behaviours compared to MoAr saline-treated rats. Finally, no motor impairment (*i.e.*, distance) was observed following MoAr and PcTx1 or mambalgin-1 administration (Fig. 2E and H).

Altogether, these results demonstrate with two different specific blockers, a clear role of ASIC1a-containing channels expressed in the BLA in pain and anxiety-related behaviours associated with arthritis.

### Injection of PcTx1 reduced arthritis-induced c-Fos expression in the BLA

**Figure 3:**
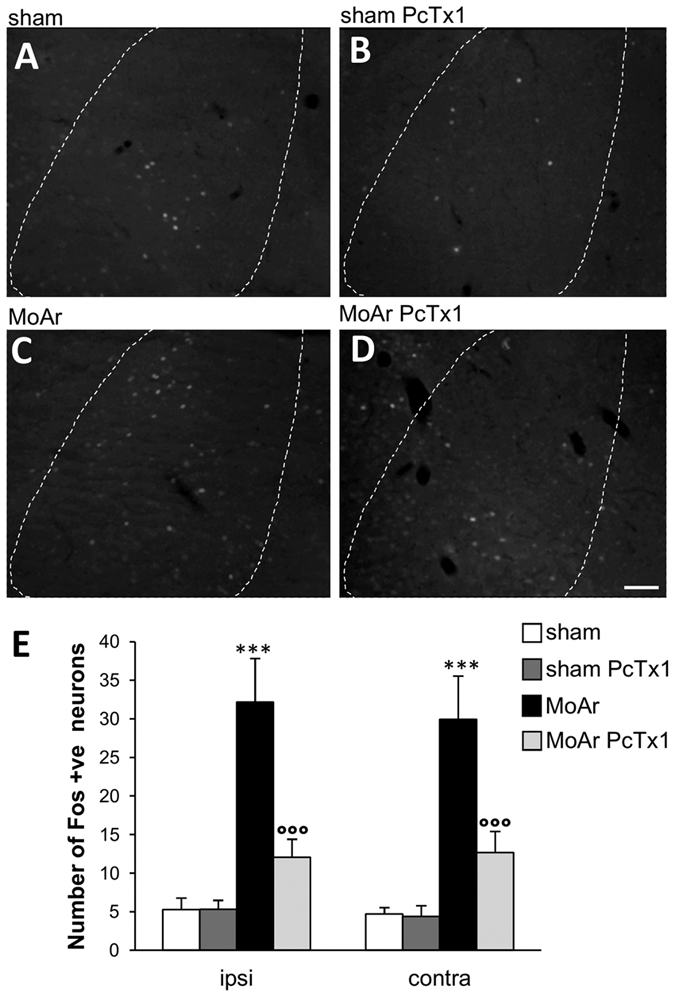
Representative photomicrographs of monoarthritis-induced upregulation of c-Fos expression in the contralateral BLA of sham pre-treated with saline (**A**) or PcTx1 (**B**), and MoAr pre-treated with saline (**C**) or PcTx1 (**D**). Scale bars = 100 μm. (**E**) Quantification of c-Fos-positive cells per section in the BLA of sham or MoAr rats as shown in (**A–D**) n = 5 animals/group (6–8 sections) for each group. One-way ANOVA followed by a Tuckey *post hoc* test. ***p ≤ 0.001 compared to sham group, °°°p ≤ 0.001 compared to Moar saline group.

We used c-Fos immunohistochemistry as an indirect marker of neuronal activity in the BLA of sham and MoAr rats treated with either saline or PcTx1. The number of c-Fos positive neurons was similar in both ipsilateral and contralateral sides of the BLA in sham rats treated with saline or PcTx1 (Fig. 3A,B and E). In contrast, a similar and bilateral increase of c-Fos expression in the BLA was observed 14 days following CFA administration (contra: 29.9 ± 5.5 and ipsi: 32.2 ± 5.6, n = 5 per group) (Fig. 3C and E). However, bilateral administration of PcTx1 in MoAr rats induced a significant decrease of c-Fos positive neurons to a level similar to the one observed in sham animals (% of reduction: ipsi 62.5 ± 4.5%, contra 57.7 ± 6.1%, n = 5 per group) (Fig. 3D and E). These results support the involvement of ASIC1a-containing channels in the neuronal response of the BLA following arthritis.

### ASIC1a is largely expressed in neurons of the BLA and its expression is not modified following CFA injection into the ankle

**Figure 4:**
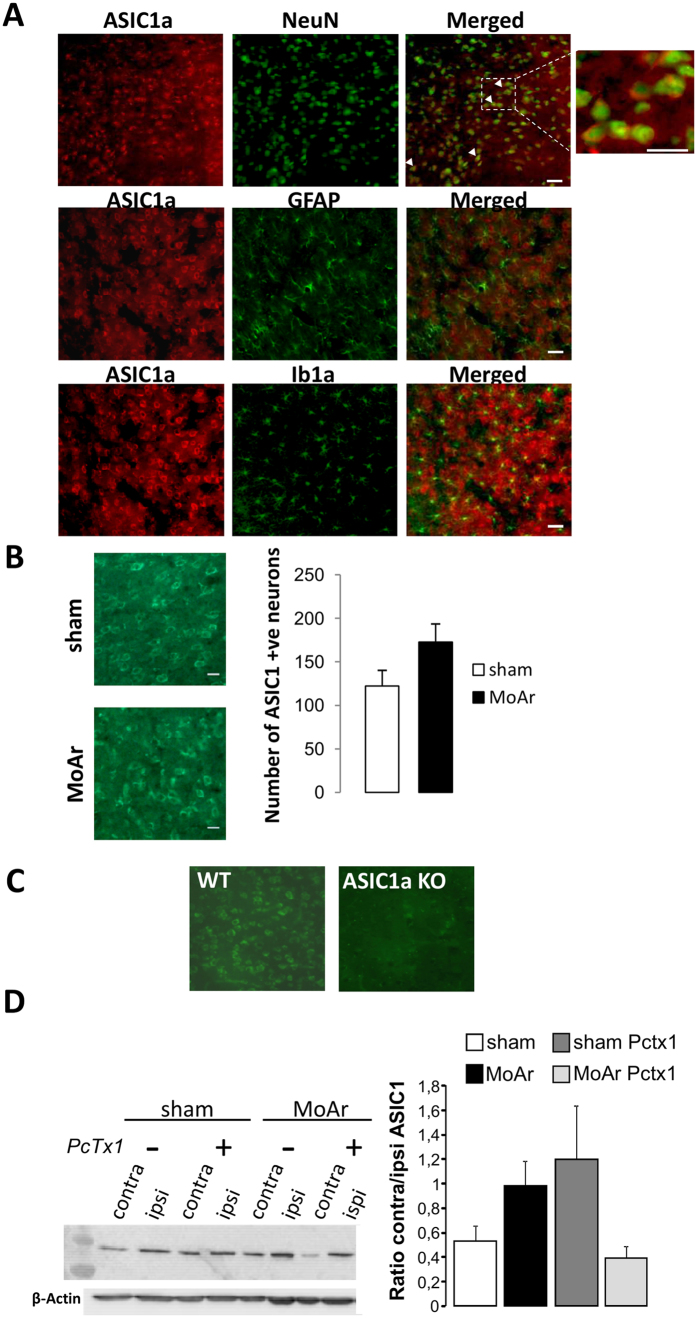
Expression of the ASIC1a protein in the basolateral amygdala (BLA) of MoAr rats. (**A**) And inset. ASIC1a (rabbit anti-ASIC1a, 1:500, alpha diagnostic) co-localized with the neuronal marker NeuN (upper panel, mouse anti-neuronal nuclei (anti-NeuN); 1:1,00) but not with an astrocyte marker (GFAP, middle panel, mouse anti-glial fibrillary acidic protein (anti-GFAP); 1:1,000) or a microglial marker (Ib1a, lower panel, rabbit anti-ionized calcium binding adaptor molecule 1 (anti-Iba1); 1:200) in MoAr rats. (**B**) Quantification of the number of ASIC1a-positive neurons in the contralateral BLA between sham and MoAr animals (n = 5 per group). (**C**) ASIC1a immunostaining (rabbit anti-ASIC1a, 1:500, alpha diagnostic) in the amygdala of wild type (left panel) and ASIC1a knockout mice (right panel). (**D**) Representative Western blot of the expression of the ASIC1a protein in ipsilateral and contralateral BLA of sham and MoAr rats treated with saline or PcTx1 (β-actin detection used for normalization shown on the bottom). Densitometric quantification of the signal corresponding to ASIC1a relative to β-actin and expressed as the ratio between the ipsi- and contralateral side for each group. n = 5 per group. One-way ANOVA followed by a Tuckey *post hoc* test.

In the contralateral BLA of MoAr rats, the ASIC1a protein colocalised with NeuN, a specific neuronal marker (Fig. 4A) without significant colocalisation with astrocyte and microglial markers (GFAP and Ib1a, respectively; Fig. 4A). We did not find a significant increase in the number of ASIC1a-positive neurons in the contralateral BLA between MoAr and sham animals (Fig. 4B). We assessed the specificity of the antibody used in the amygdala of ASIC1a knockout mice where we did not observed any significant staining compared to WT animal (Fig. 4C). We further investigated the expression level of ASIC1a using punch biopsies of the ipsilateral and contralateral sides of the BLA of sham and MoAr rats treated with saline or PcTx1. Western blot analysis did not show any significant change of the ASIC1a expression in the contralateral side of the BLA of sham saline-treated rats (Fig. 4D). Therefore, CFA administration did not increase ASIC1a expression in the BLA at D14. Importantly, bilateral administration of PcTx1 did not induce any significant change in MoAr group (Fig. 4D).

### Expression profile of ASIC and other genes in the basolateral complex of the amygdala in sham and monoarthritic animals

**Figure 5:**
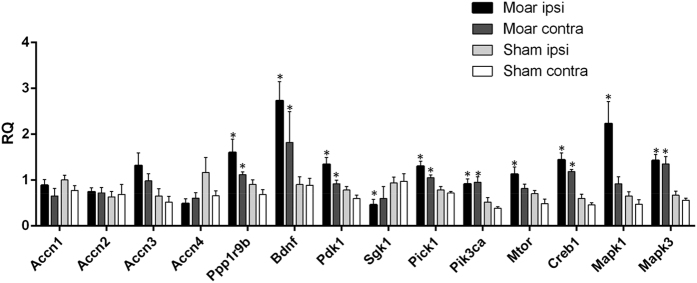
Gene expression profiling in the amygdala. Relative quantity (RQ) histogram of the transcripts differentially expressed in the ipsilateral and contralateral basolateral complex of the amygdala between sham and MoAr rats assessed using TLdA. *p ≤ 0.05 between the RQ of the ipsi or contra MoAr groups respective to the sham side (i.e., ipsi or contralateral sides respectively (non parametric Kruskall-Wallis test)). n = 6 per group (in duplicate).

To further investigate the effect of arthritis on the expression of ASIC and other genes of potential interest for neurotransmission/plasticity, 39 genes were analyzed using custom-made TaqMan^®^ Low Density Array for gene expression profiling based on qPCR (supplementary Table 1). Gene expression was assessed in the ipsilateral (right) and contralateral (left) sides of the basolateral complex of the amygdala from sham and MoAr animals. As shown in supplementary Table 1 and Fig. 5, the expressions of Accn1, 2, 3 and 4 genes encoding the different subunits of ASIC channels, showed no significant variation between ipsilateral and contralateral sides (relative to the lesion) in sham, MoAr, and also between MoAr and sham animals (Fig. 5), in agreement with the previous data obtained for expression of the ASIC1a protein (Fig. 4B and D).

There was no significant change of markers of plasticity such as Arc, Ncam1, Gap43, Dlg4, Syp and Egr1. However, the expression of Ppp1r9b (spinophilin), a protein phosphatase, was significantly increased in both sides of the amygdala in Moar rats compared to sham (160% and 178% respectively, p ≤ 0.05, Fig. 5). More interestingly, we observed a significant increase of Bdnf mRNA level in the contralateral and ipsilateral sides in MoAr rats compared to the respective side of sham (197% and 299% respectively, p ≤ 0.05, Fig. 5). Moreover, the expression level of PIK3CA encoding PI3K, a protein kinase downstream to BDNF signalling and involved in the regulation of spinal ASIC1a channels expression^14^, was significantly increased in the contralateral and ipsilateral sides of MoAr compared to the respective side of sham animals (255% and 178% respectively, p ≤ 0.05, Fig. 5)^14^,^23^. The expression level of several others kinases were also increased in MoAr rats compared to sham. Sgk1 mRNA, which is a negative modulator of ASIC1a channel expression, exhibited a significant reduction in the ipsilateral side of MoAr rats compared to sham animals^24^. In contrast, the expression of PICK1, which is a positive regulator of ASIC1 channel expression, was enhanced in both sides of the BLC of MoAr rats compared to sham (151% and 171% in the contralateral and ipsilateral sides, respectively, p ≤ 0.05, Fig. 5 25,26). Thus, it seems that the expression of some of the factors identified as regulators of ASIC1a channel expression/function was disrupted in the amygdala of MoAr rats compared to sham. Other kinases such as Mtor mRNA encoding mTor protein kinase only displayed an increase expression of 161% in the ipsilateral side in MoAr rats compared to sham. The gene expression of Mapk1 encoding for ERK2 protein kinase, which have been shown to have an important role in the modulation of the perception of pain in the amygdala^27^, was only increased in the ipsilateral side of MoAr rats compared to the ipsilateral side of sham (348%, p ≤ 0.05, Fig. 5), while the mRNA level of MapK3 encoding ERK1 was significantly increased in both sides of the BLC of MoAr rats compared to sham group (242% and 217% in the contralateral and ipsilateral sides, respectively, p ≤ 0.05, Fig. 5). Finally, expression of Creb1 and Pdk1 mRNA were significantly increased in both sides of MoAr rats compared to sham (supplementary Table 1 and Fig. 5).

The expression of Gria1 and Gria2 encoding the glutamate receptors GluR1 and GluR2, as well as NMDA subunits (NR1, NR2A and NR2B) was relatively unaffected by arthritis. It is important to note that Gria1, Gria2, Grin2a and Grin2b expressions were increased in the ipsilateral side of MoAr rats but they failed to reach statistical significance.

Concerning genes coding for precursor of opioidergic peptides (Penk encoding for proenkephalin, POMC, the precursor of β-endorphin and Pdyn, precursor of dynorphin), their expression was relatively unchanged between groups except for Penk, which was reduced in the amygdala of MoAr rats without reaching statistical significance (−82%, ipsilateral side of MoAr rats, p = 0.052, Fig. 5). Finally, expressions of OPRM1, OPRD1 and OPRK1 genes encoding μ, δ and κ opioid receptors, respectively, were not altered in any groups.

### The level of phosphorylated Akt increased in monoarthritic rats and is reduced by intra-BLA injection of PcTx1

**Figure 6:**
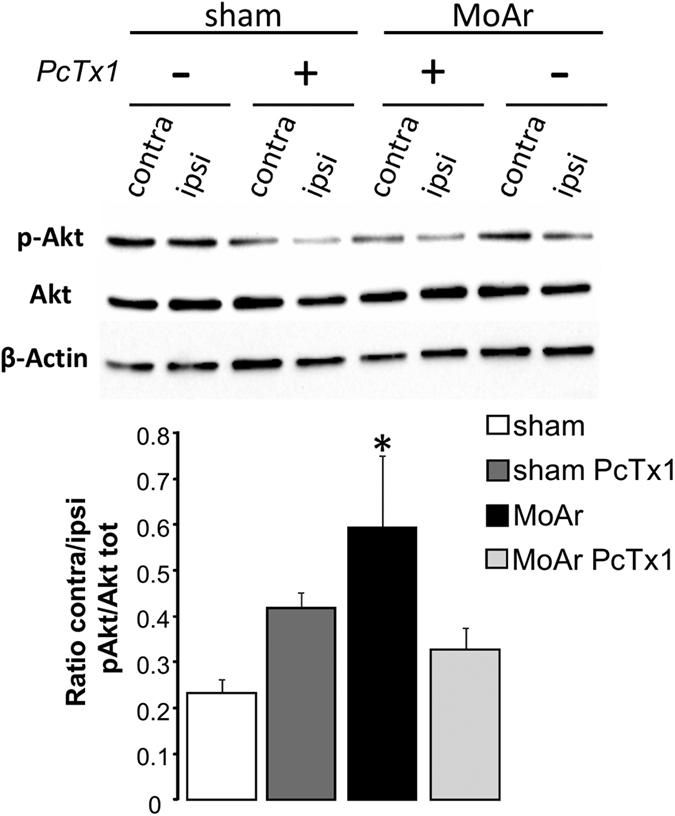
Expression of phospho Ser473-Akt and total Akt proteins in ipsilateral and contralateral BLA of sham and MoAr rats treated with saline or PcTx1 evaluated by Western blot. Representative Western blot (upper panel) and quantification of phospho-Akt and total Akt of sham and MoAr rats treated with saline or PcTx1 (lower panel). All phospho bands of p-Akt were normalized relative to total Akt protein and expressed as the ratio between the ipsi- and contralateral side for each group. n = 5 per group. One-way ANOVA followed by a Tuckey *post hoc* test. *p ≤ 0.05 compared to sham saline group.

Expression data showed a significant increase of the level of transcripts encoding PI3K in MoAr animals. The PI3-kinase/Akt pathway was previously described to be involved in BDNF-induced pain hypersensitivity at the spinal level through ASIC1a channels^8^. We therefore looked at this pathway as a potential regulator of ASIC1a expression in the BLA of MoAr rats. Expression of phospho Ser473-Akt and total Akt proteins was analyzed by Western blot in contralateral and ipsilateral BLA of sham and MoAr rats treated with either saline or PcTx1 (Fig. 6). The contra/ipsi expression ratio of pAkt/totalAkt in the BLA was unchanged between both sides in sham group treated with either saline or PcTx1 (Fig. 6). However, this ratio was significantly increased in MoAr saline-treated rats compared to sham saline-treated animals indicating that there is a significant increased in the expression of p_-_Akt in the contralateral BLA of MoAr rats compared to sham rats (Fig. 6). Interestingly, the bilateral administration of PcTx1 in BLA prevented this increase in MoAr animals.

## Discussion

This study provides the first evidence of a key role of neuronal ASIC1a expressed in the BLA in pain and anxiety behaviours associated with arthritis. Intra-BLA administration of two independent specific blockers of ASIC1a-containing channels, PcTx1 and mambalgin-1, both reduced pain as well as anxiety related behaviours associated with arthritis without any significant effect in sham animals. The effect of PcTx1 was correlated with a reduction of the increase of neuronal activity within the BLA evaluated by cFos immunohistochemistry. Interestingly, we did not observed any significant changes in ASIC subunits mRNA and total ASIC1a protein expression following arthritis, likely suggesting an alteration in the trafficking and/or electrophysiological properties of ASIC1a channels in the BLA. In addition, assessment of the expression of different factors in the basolateral complex of the amygdala of sham or MoAr rats using TaqMan RTqPCR revealed significant changes in the mRNAs expression of a marker of plasticity (Ppp1r9b), the neurotrophic factor BDNF, and several kinases (Pdk1, Sgk1, Pick1, PI3K, Mtor, Creb1, Mapk1/3), between MoAr rats and sham animals. Interestingly, the level of phosphorylated Akt was increased in MoAr rats, which may be consistent with a potential role of the BDNF/PI3K/Akt pathway in the regulation of ASIC1a, as previously described following spinal BDNF administration^14^.

The amygdala has emerged as a crucial player in the sensory-discriminative and the emotional/cognitive aspects of pain in animal and human^4^,^5^,^28^,^29^. For example, fMRI studies has demonstrated that the amygdala is involved in the so called “pain matrix” in several pain disorders including rheumatoid arthritis^30^,^31^,^32^. The basolateral nuclei of the amygdala, beyond its role as a mere input provider to the central and lateral bed nucleus of the stria terminalis, also appears as an important factor in pain processes and associated phenomenon such as neuronal plasticity^8^,^11^,^33^. ASIC1a is robustly expressed in the lateral and basolateral nuclei of the amygdala^17^,^23^, and overexpressing ASIC1a within the amygdala enhances contextual fear conditioning in resting conditions^34^. Importantly, a contribution of post-synaptic ASIC currents to synaptic plasticity was recently demonstrated in the lateral amygdala^17^. Finally, functional plasticity of BLA neurons characterized by an increased in spontaneous and evoked activity and enhanced synaptic transmission has been observed in an arthritic rat model^28^. Here, we demonstrated that inhibition of ASICs channels by local injection in the BLA of two potent and specific inhibitors of ASIC1a-containing channels, PcTx1 or mambalgin-1, reduces mechanical and thermal hyperalgesia but also anxiety related behaviours (*i.e.*, elevated plus maze and social interaction test) associated with arthritis. Both inhibitors have a similar effect, which suggests based on the overlap of their respective pharmacological profiles^35^, a major contribution of ASIC1a homomeric and/or ASIC1a/ASIC2b heteromeric channels in this effect, and therefore a minor contribution of ASIC1a/ASIC2a heteromeric channels blocked by mambalgin-1 but not PcTx1. This is further supported by the fact that injection of the same concentration of both inhibitors led to a slightly more potent effect of PcTx1, which blocks more efficiently ASIC1a and ASIC1a/ASIC2b channels than mambalgin-1^35^. Further experiments specifically targeting ASIC2 subunit using viral vector should help to understand the specific role of this particular subunit. We observed an increased neuronal activity in the BLA of MoAr rats, which was significantly reduced by PcTx1 administration. An increased of neuronal activity in the amygdala, including the BLA, has been already observed in several studies, directly by electrophysiological approaches or indirectly, using markers such as c-Fos^36^,^37^,^38^,^39^. However, to our knowledge, this is the first time that a link between ASIC1a channels expressed in the amygdala and pain as well as anxiety associated with chronic pain is demonstrated, which seems to be related to increased neuronal activity.

The ASIC1a protein is mainly, if not exclusively, expressed in neurons and not glial cells of the BLA regardless of the condition (sham or MoAr animals), and its expression, assessed by Western blot, is not different in MoAr animal compared to the sham group. These results imply that changes in the functional properties of ASIC1a and/or its trafficking to the neuronal plasma membrane may occur in the BLA of MoAr rats, similarly to what has been described in the spinal cord by Duan *et al*.^14^. These authors have demonstrated that ASIC1a underlined spinal BDNF-induced pain hypersensitivity through a mechanism of spinal central sensitisation involving the PI3-kinase/Akt pathway. They established that activation of the PI3-kinase/Akt pathway by BDNF regulates ASIC1a membrane insertion, ultimately increasing its function. Strikingly, we observed a significant increase of Bdnf mRNA, PI3-kinase mRNA level and phosphorylated Akt in the BLA of MoAr rats compared to sham animals. However, this increased expression of p-Akt is reduced by the administration of PcTx1, which may argue against an upstream regulation of ASIC1a channel or might suggest a negative feedback loop. Although this needs further investigation, these results highlight a potential role of the PI3-kinase/Akt pathway in the regulation of ASIC1a in BLA neurons, certainly in a BDNF-dependent manner. Indeed, a rise of the Bdnf mRNA level was observed specifically in the BLA of MoAr rats. A supraspinal role of BDNF in the sensory-discriminative and emotional aspect of inflammatory pain has been already demonstrated in others brain nuclei such as the primary sensory cortex and the cingulate cortex^40^. Furthermore, increased expression of BDNF and its cognate receptor, TrkB, was demonstrated within the amygdala in a model of neonatal maternal separation induced visceral pain^41^. Such a mechanism could contribute to the sensitisation of the BLA-CeA synapse involved in chronic pain and associated anxiety^5^,^42^,^43^. The increase expression of Ppp1r9b (*i.e.*, spinophilin) in the BLA indicates that synaptic plasticity occurred in our arthritic model^44^, which needs to be confirmed by electrophysiological experiments. To reinforce this hypothesis, it has been recently demonstrated that (i) presynaptic stimulation increases extracellular protons released from the lateral amygdala neurons and activates ASICs, (ii) the high frequency stimulation-induced LTP in lateral amygdala is drastically reduced in ASIC1a knockout animals, and (iii) acidic puffs induce LTP in lateral amygdala neurons of WT but not ASIC1a knockout animals^17^. Taken together, it is plausible that, in a chronic pain context, increased synaptic input in the amygdala due to peripheral afferent barrage, increases post-synaptic ASIC-dependant currents mainly involving ASIC1a-containing channels. Interestingly, pre-LTP has been recently shown to occur in the ACC, where it may converge with post-LTP to mediate the interaction between pain and associated anxiety^45^. It will be interesting to determine if a similar mechanism also occur in the amygdala.

Analysis of the expression of different factors in the basolateral complex of the amygdala of sham or MoAr animals using TaqMan RTqPCR card also revealed significant changes in the mRNAs expression of others kinases beyond PI3K (*i.e.*, Pdk1, Sgk1, Pick1, Mtor, Creb1, Mapk1/3) in MoAr rats. Interestingly, some of these kinases, especially Pick1 and Sgk1, are potential modulators of ASIC1 expression and/or function. A brain-specific Sgk1 splice isoform is able to diminish the expression of ASIC1 at the plasma membrane, reducing ASIC1 function^24^. The decrease of Sgk1 expression in MoAr rats might suggest an enhanced expression of ASIC1 at the plasma membrane. This phenomenon could be reinforced by the increased expression of Pick1 since it plays a key role in the regulation of ASIC functions^25^,^26^. In cortical neurons from PICK1-KO mice, both the amplitude of ASIC currents and the elevation of intracellular calcium by acid are reduced, which were attributable to decreased expression of the ASIC1a and ASIC2a proteins in the plasma membrane^25^. Conversely, Pick1 overexpression enhanced ASIC1a cell surface expression^26^. Thus, some known negative or positive regulators of ASIC1 expression were decreased or increased, respectively, in MoAr rats, which could lead to an enhanced membrane expression of neuronal ASIC1a. However, no significant change in the mRNA expression of the different ASICs subunits, including ASIC1a, has been detected following arthritis, which is consistent with the protein expression data. If only the membrane expression of neuronal ASIC1a is enhanced in the amygdala, it could not be detected in the total protein/mRNA extract. Finally, an increase in the expression of Creb1, Mapk1 and Mapk3 mRNAs has been found. However, there was no significant difference in the expression of phosphor-ERK1/2, using western blot, between groups despite an increase in the contralateral side of the BLA in Moar saline treated group (supplementary Figure 4). This result could provide further evidence that the PI3K/Akt pathway within the BLA is more likely contributor in ASIC1a involvement. Several studies have demonstrated that activation of ERK in the amygdala contribute to pain-related synaptic facilitation and pain behaviour in inflammatory arthritis or formalin pain models, or in a model of acid induced muscle pain^27^,^46^,^47^. While blocking ERK did not affect responses to acute noxious stimuli in the absence of inflammation, it reduced synaptic plasticity, audible and ultrasonic vocalisations and spinal reflexes of arthritic rats^47^. Increased CREB expression, especially the phosphorylated form, could also participate in amygdala sensitisation and pain^48^.

In conclusion, this study provides the first evidence of a role of neuronal ASIC1a-containing channels in the basolateral amygdala in pain behaviours and anxiety associated with arthritis. The underlying mechanism requires further investigation but may involve the BDNF/PI3K/Akt pathway as previously shown in the spinal cord. Taken together, these results reinforce the interest of ASIC1a channels as target for chronic pain treatment.

## Materials and Methods

### Rat monoarthritis model

Male Sprague-Dawley rats (150–175 g, Janvier, France) were acclimatized for a week before testing. Rats were housed in a controlled room with a 12-hour light/dark cycle, with free access to food and water. Rats were housed 4 per cage except after the bilateral implantation experiment (2 per cage and individually following surgery). Monoarthritis (MoAr) was induced by a single unilateral intra-articular injection of 50 μl complete Freund’s adjuvant (CFA, heat-killed *Mycobacterium* 2 mg/ml; Difco Laboratories, Detroit, MI, USA) in the right hindpaw under isoflurane (4%) anaesthesia as previously described by ref. 49. Sham rats were injected with 50 μl of vehicle in the same conditions. Behavioural experiments were performed 14–16 days after monoarthritis induction, when rats weighed between 260 to 320 g, to respect stereotaxic coordinates from^50^. All procedures were approved by the local ethical committee (Comité Régional d’éthique en matière d’expérimentation animale Auvergne (CEMEA-Auvergne), protocols numbers: CE12–10 and CE21–10) and followed guidelines of the International Association for the Study of Pain and ARRIVE^51^,^52^.

### Implantation and microinjection of PcTx1 or mambalgin-1 into the basolateral amygdala of sham and MoAr rats

One week after monoarthritis induction, rats were deeply anesthetised with a mixture of xylazine hydrochloride (Rompun^®^, 10 mg/kg) and ketamine (Imalgene 500^®^, 80 mg/kg). Stainless steel 26-gauge guide cannulas (Plastic One, Roanoke VA, USA) were both stereotaxically implanted into the BLA ((Bregma: AP = −2.8 mm, L =  ± 4.8 mm, DV = −7.6 mm)^50^). Guides were affixed onto the skull with photopolymerised cranioplastic cement (Optibond, Henri Schein, France). Until the microinjections, guide cannulas were occluded with dummy cannulas (Plastic One, Roanoke VA, USA) to maintain their patency. At least 7 days after the surgery (*i.e.*, 14–16 days following CFA administration), bilateral microinjections were performed through injection cannulas, (33-gauge, Plastic One, Roanoke VA, USA) that extended 1 mm beyond the guide cannula, connected to an Hamilton syringe preloaded with specific ASIC1a-containing channel peptide inhibitors, *i.e.*, synthetic PcTx1 (0.05 μg-11 pmoles/side; Synprosis/Provepep, Fuveau, France), a specific blocker of homomeric ASIC1a and probably heteromeric ASIC1a/ASIC2b channels^21^,^53^, or synthetic mambalgin-1 (0.065 μg–10 pmoles/side; Smartox Biotechnology, Grenoble, France), another specific inhibitor with a broader pharmacological profile than PcTx1 including homomeric ASIC1a and heteromeric ASIC1a/ASIC2b plus ASIC1a/ASIC2a channels^13^,^22^, or saline. Compounds were manually delivered (0.5 μl over 60 s) into the BLA in moderately anesthetized rats (1.5% isoflurane inhalation). The injection cannula was left in place for an additional minute to prevent any drug backflow. At the end of the pharmacological experiments, when rats were killed for immunohistochemistry or biopsie punch, the needle track was verified under a binocular microscope to confirm the infusion site. After this examination, 9 rats from different groups were found to have at least one infusion needle terminating outside the BLA and were therefore excluded from subsequent analysis (see Supplementary Figures 2 and 3 for detailed implantation).

### Pharmacological experiments

The effect of a bilateral intra-BLA administration of saline or PcTx1 (0.05 μg/0.5 μl/side) 15 min before the behavioural tests was evaluated in sham and MoAr rats, 14 to 16 days following monoarthritis induction. The behaviour of each rat was then assessed in the different tests (*i.e.*, social interaction or elevated plus maze for anxiety, and paw pressure or paw immersion for pain, see below). To confirm the specificity of the effect observed following bilateral intra-BLA administration of PcTx1, we also assessed the effect of bilateral intra-BLA administration of saline or mambalgin-1 (0.065 μg–10 pmoles/side), 15 min before paw pressure test for pain and the elevated plus maze for anxiety, in sham and MoAr rats, 14 to 16 days following monoarthritis induction.

### Measurement of mechanical hypersensitivity

Mechanical hypersensitivity in sham and MoAr animals was assessed with an analgesiometer as previously described (paw pressure test, Ugo Basile, Varese, Italy^54^). Nociceptive thresholds were measured by applying increased pressure to the ipsilateral ankle of unrestrained rats until a vocalisation was obtained^54^. The analgesiometer could be used to assess withdrawal, which is considered to be spinally mediated and as any withdrawal reflex could be affected by several factors such as motor impairment. Vocalisation threshold, which is considered to be more supraspinally mediated and integrated, seems more accurate to assess pain behaviour and sensitivity to analgesic treatments. However, it could have been interesting to also assessed evoked withdrawal behaviours.

Treatments have been administered after measurement of two consecutive stable nociceptive (*i.e.*, vocalisation) threshold values that differ by not more than 10%, with at least a 10 min interval between two measurements. Vocalisation threshold were assessed at baseline pre and post-CFA and 15, 25 and 45 min following PcTx1, mambalgin-1 or saline administration.

### Measurement of thermal hypersensitivity

Thermal hypersensitivity in MoAr and sham animals was assessed using ankle/paw -immersion test in water maintained at 46.0 ± 0.2 °C using a thermoregulated water bath. The ankle/paw of rat was immersed in water until the paw is withdrawn. The duration of ankle/paw immersion was recorded, and a cut-off time of 25 s was used to avoid any tissue damage. Rats have been habituated to the testing procedures and to handling by the investigator during the week prior to the experiment. Treatments have been administered after measurement of two consecutive stable withdrawal threshold values that differ by not more than 10%, with at least a 10 min interval between two measurements. Paw withdrawal threshold were assessed at baseline pre- and post-CFA, 15 and 45 min following PcTx1 or saline administration.

### Social interaction test

Pairs of rats from different cages but similarly treated (*i.e.*, pairs of sham or MoAr rats treated with saline or PcTx1) were placed in an open arena (75 × 75 × 40 cm) in grey Plexiglas under dim light conditions (30 lux) for 10 min following a 30 min period of isolation. Social interaction^55^ defined by the time spent sniffing, following, grooming, boxing and wrestling between pairs was scored manually. A decrease in social behaviour (exploration of a new congener) indicates increased anxiety.

### Open field test

The open field test took place in the same arena used for the social interaction test, under dim light conditions (30 lux).The distance travelled and the velocity were automatically calculated during the 5-min testing period (Ethovision, Noldus) in order to eliminate motor impairment following CFA injection and/or PcTx1 infusion.

### Elevated plus maze test

The elevated plus maze (EPM) consists of four arms, two opposite open arms (50 × 10 × 10 cm) and two opposite closed arms (50 × 10 × 10 cm), joined by a common central platform (10 cm × 10 cm), subjected to an equal illumination (30 lux). The maze was elevated to 60 cm above the floor. Each animal was placed into the EPM facing an open arm. The time spent in open and closed arms, the number of entries into the open and closed arms (considered when head, gravity center and tail points are located within the arm) as well as distance travelled and velocity were automatically recorded for 5 min (Ethovision, Noldus). The percentage of the time spent into the open arms and the percentage of number of entries into the open arms were calculated for each animal.

### TaqMan^®^ Low Density Array

Following assessment of mechanical hypersensitivity at day 14 following CFA or vehicle administration, rats were sacrificed by decapitation. Brains were then quickly dissected out and coronal sections were obtained using a 1.0 mm rat brain slicer (Phymep, France). The amygdala structure was removed on ice and a biopsy punch of 2 mm of the basolateral complex (BLC) was taken from the ipsilateral and contralateral side from sham and MoAr animals (coordinates of the coronal slices in mm relative to bregma [−2; −4]ref. 50). The sampling process lasted less than 5 min and samples were rapidly frozen in liquid nitrogen and stored at −80 °C to avoid any degradation.

Quantitative real-time PCR analysis was performed with custom designed arrays in 384-well microfluidic cards TaqMan^®^ Low Density Array (TLdA; Applied Biosystems, France). Each well contains specific, user-defined primers and 6-FAM labeled TaqMan MGB probes (6-carboxyfluorescein as FAM and dihydrocyclopyrroloindole tripeptide minor groove binder as MGB), capable of detecting a single gene. The card was configured into 4 identical 39-genes sets (8 samples per card in duplicate) including two housekeeping genes, rplp2 and 18 S. Thirty nine genes were chosen from the literature for their role in anxiety associated to chronic pain in the amygdala, their possible interaction with ASIC1a (Pick1 and Sgk1 for example) or their involvement in synaptic plasticity and/or potentiation (spinophilin, Erg1, AMPA, NDMA subunits (supplementary Table 1)). A total of 100 μl reaction mixtures with 50 μl cDNA templates (100 ng) and an equal volume of TaqMan^®^ universal master mix (Applied Biosystems, France) was added to each line of TLdA after gentle vortex mixing. The array was centrifuged twice at 331 × g for 1 min to distribute the samples from the loading port into each well. The card was then sealed and introduced into Applied Biosystems Prism 7900HT sequence detection system according to recommended thermal cycling conditions for microfluidic cards (2 min at 50 °C, 10 min at 94.5 °C and 30 s at 97 °C, and 1 min at 59.7 °C for 40 cycles). The threshold cycle (Ct) was automatically given by SDS v2.2 software package. Relative quantities (RQ) were determined using the equation: RQ = 2^−ΔΔCt^ 56. Rplp2 and 18 S were used as housekeeping genes. The table data is presented as a percentage of variation of the log base 2 (Log2) of normalized fold change relative to the comparison of the different conditions (*i.e.*, ipsilateral and contralateral, sham or MoAr rats). We used the Log2 to allow visualization of the fold change in the positive (upregulated) and negative (downregulated) way where Log2 of 0 equals no change in gene expression (0%) and 1 (100%) and −1 (−100%) equal two fold up- and down-regulation, respectively.

### Immunohistochemistry for c-Fos and ASIC1a

At the end of the behavioural experiments (60 min after saline or PcTx1 administration), MoAr and sham animals (n = 5 in each group) injected with PcTx1 or saline were terminally anaesthetised using pentobarbitone and quickly perfused transcardially with saline followed by 4% paraformaldehyde (PFA). After perfusion, the brain was excised, post-fixed for 24 hours in the same perfusion fixative, cryoprotected in 30% sucrose in 0.1 M phosphate buffer (PB) for 48 hours at 4 °C, and then frozen in OCT. Transverse sections (30 μm) were cut on a cryotome (Microm HM450, Thermo Scientific). Free floating sections of the amygdala were stained for c-Fos immunohistochemistry as follows: after 3 washes in TBS 0.05 M pH 7.6, sections were incubated for 1 hour in a blocking solution (TBS 0.05 M, BSA 3%, Triton 0.4%, Donkey serum 1%) and then overnight at 4 °C with a rabbit primary antibody anti-Fos (1:7,000 in TBST; Santa Cruz, USA). After 3 washes in TBS 0.05 M, sections were incubated for 2 hrs with the appropriate secondary antibody (AlexaFluor^TM^ 488 goat anti-rabbit IgG; 1:1,000; Molecular Probes, USA). Sections were then washed in TBS 0.05 M, mounted on gelatine coated slides and cover-slipped with Dako fluorescent mounting medium. From each animal, 4–6 sections were randomly selected for counting c-Fos positive cells in the ipsilateral and contralateral sides of the BLA (n = 5 per group) by a blinded investigator and an average of these counts was taken.

To determine the cellular distribution of ASIC1a, sections from MoAr rats were double stained with primary antibody against ASIC1a (rabbit anti-ASIC1a, 1:500, alpha diagnostic) and primary antibodies against markers for neurons [mouse anti-neuronal nuclei (anti-NeuN); 1:1,000; Millipore], astrocytes [mouse anti-glial fibrillary acidic protein (anti-GFAP); 1:1,000; Chemicon International], followed by the appropriate secondary antibody solution ([donkey anti-rabbit IgG-conjugated AlexaFluor^TM^ 488; 1:1,000; Molecular Probes, USA for ASIC1a or [goat anti-rabbit IgG-conjugated Alexa Fluor 546^TM^; 1:1,000; Molecular Probes, USA]) for NeuN and GFAP. For counterstaining of the microglial cell population Ib1a staining [rabbit anti-ionized calcium binding adaptor molecule 1 (anti-Iba1); 1:200; Wako Pure Chemical Industries Ltd, Japan] was visualized with ExtrAvidin-FITC (1:500, Sigma) following signal amplification with ABC (Vector Laboratories) and biotinyl tyramide (NEN Life Science Products).

To determine the number of ASIC1a positive neurons, free floating sections of sham saline and MoAr saline rats were stained as described above with the primary antibody against ASIC1a (rabbit anti-ASIC1a, 1:500, alpha diagnostic) followed by the appropriate secondary antibody solution [donkey anti-rabbit IgG-conjugated AlexaFluor^TM^ 488; 1:1,000; Molecular Probes, USA). From each animal, 4–6 sections were randomly selected for counting ASIC1a positive cells in the contralateral sides of the BLA (n = 5 per group) by a blinded investigator and an average of these counts was taken.

To assess the specificity of the antibody used, free floating sections of the amygdala from perfused knock-out ASIC1a and wild type mice were stained as described above with the primary antibody against ASIC1a (rabbit anti-ASIC1a, 1:500, alpha diagnostic) followed by the appropriate secondary antibody solution (donkey anti-rabbit IgG-conjugated AlexaFluor^TM^ 488; 1:1,000; Molecular Probes, USA).

Sections were visualised under Nikon Eclipse Ni-E fluorescent microscope with Nikon analysis software (NiS element).

### Western immunoblotting

At the end of one pharmacological experiment (45 min after saline or PcTx1 administration), sham or MoAr rats of the different groups (n = 5 in each group) were terminally anesthetized with pentobarbitone, sacrificed by decapitation and fresh biopsy punch (1 mm) from ipsilateral and contralateral basolateral amygdala were dissected out and snap frozen in liquid nitrogen. Tissue samples were subsequently homogenized in RIPA (RadioImmunoPrecipitation Assay) buffer (50 mM Tris HCl pH 7.5, 150 mM NaCl, 1 mM EDTA, 1% NP-40, 0.1% SDS + 0.5% Deoxycholic acid containing Complete protease inhibitor cocktail) using a glass homogenizer. Homogenates were then centrifuged at 14,000 × g for 10 min at 4 °C and supernatants were collected. BLA amygdala whole cell lysates were next titrated to determine their protein concentrations using a BCA Protein Assay kit (Pierce, UK).

For Western immunoblotting, lamaelli loading buffer was added to the samples containing equal weights of total proteins (30 μg) and heated at 70 °C for 30 min. The samples were separated by denaturing and reducing sodium dodecyl sulphate polyacrylamide gel electrophoresis (SDS-PAGE) using 7.5% acrylamide gels. The separated proteins were then transferred to nitrocellulose membranes using Bio-Rad wet blotting system.

All membrane incubations were performed on a rotating plate. Washes were done in TBS-T (50 mm Tris-HCl and 6 mM NaCl containing 0.1% Tween 20). Blocking of unspecific binding sites was done by incubating the membranes in 5% non fat dry milk in TBS-T for 1 hour at room temperature. The membranes were then incubated with the primary antibodies diluted in 5% non fat milk in TBS-T overnight at 4 °C. After washing, the membranes were probed with appropriate Horseradish peroxidase conjugated secondary antibody diluted at 1:10,000 with 5% non fat dry milk in TBS-T for 1 hour at room temperature. Antibodies to pAKt (ser473, 1:1,000; catalog #9271), phospho-p44/p42 MAPK (ERK1/2), (1:1,000; catalog #4976), total Akt (1:1000; catalog #9272) were obtained from Cell Signalling Technology. Anti-ASIC1a (1:1,000) and β-actin (1:5,000) were obtained from Alpha diagnostic and Sigma, respectively. The specificity of ASIC1a antibody was verified by incubated with the control/blocking peptide (Alpha diagnostic, data not shown).

The immunoreactive bands were quantified by densitometric analysis using Bio-Rad imaging software (Chemidoc). The band analysis was accomplished by capturing the corresponding relative intensities using the imager ChemiDoc XRS running Chemidoc imaging software (BioRad, St Louis, USA), which were corrected by subtracting the background. The Phospho band intensities of pAkt were normalized relative to those of total pAkt bands and the ASIC1a or phospho-ERK1/2 band intensities were normalized relative to those of β-actin bands. Results are expressed as the mean ± S.E.M of expression levels of the ratio between contralateral and ipsilateral BLA for each group (both ipsilateral and contralateral protein samples were run on the same gel).

### Data analyses and statistics

Results are presented as mean ± standard error to the mean (S.E.M). The Percentage Maximum Possible Effect (%MPE) was calculated as the percentage difference between the measured response at a given time and the baseline response, divided by the difference between the cut off for each pain tests (*i.e.* 25 s and 250 g for the thermal and paw pressure test, respectively) and the baseline response. For the TLdA experiment, statistical comparisons between the ipsilateral and contralateral sides from sham and MoAr groups were performed using the non-parametric Kruskall-Wallis test. A one-way analysis of variance (ANOVA) was used to compare more than two groups. When sham group received the same treatment than MoAr group, a two-way ANOVA was used. When the F value was significant, this was followed by Tukey test for multiple comparisons (Sigmastat 2.03). In all cases, the significance level was set at p < 0.05.

## Additional Information

**How to cite this article**: Aissouni, Y. *et al*. Acid-Sensing Ion Channel 1a in the amygdala is involved in pain and anxiety-related behaviours associated with arthritis. *Sci. Rep.* **7**, 43617; doi: 10.1038/srep43617 (2017).

**Publisher's note:** Springer Nature remains neutral with regard to jurisdictional claims in published maps and institutional affiliations.

## Supplementary Material

Supplementary Figures

Supplementary Dataset 1
