# Supplementary material for: Acid-Sensing Ion Channel 1a in the amygdala is involved in pain and anxiety-related behaviours associated with arthritis
Source: Sci Rep. 2017 Mar 2;7:43617. doi: 10.1038/srep43617 (PMC5340794; doi:10.1038/srep43617)
Supplement: Supplementary Figures [file srep43617-s1.pdf]

**Acid-Sensing Ion Channel 1a in the amygdala is involved in pain and anxiety-related behaviours associated with arthritis**

Youssef Aissouni, Abderrahim El Guerrab, Al-Madhy Hamieh, Jeremy Ferrier, Maryse Chalus, Diane Lemaire, Stéphanie Grégoire, Monique Etienne, Alain Eschalier, Denis Ardid, Eric Lingueglia ,Fabien Marchand

**Supplementary Table 1:** Differentially expressed genes in ipsilateral and contralateral basolateral complex of the amydala of monoarthritic rats compared to controls using TLdA. Data are expressed as fold changes where 0 equals no change in gene expression and 1 and -1 equal two fold up- and down regulation, respectively. Non-parametric Kruskall-Wallis test. Significant differences are in bold.

**Acid-Sensing Ion Channel 1a in the amygdala is involved in pain and anxiety-related behaviours associated with arthritis**

Youssef Aissouni, Abderrahim El Guerrab, Al-Madhy Hamieh, Jeremy Ferrier, Maryse Chalus, Diane Lemaire, Stéphanie Grégoire, Monique Etienne, Alain Eschalier, Denis Ardid, Eric Lingueglia ,Fabien Marchand

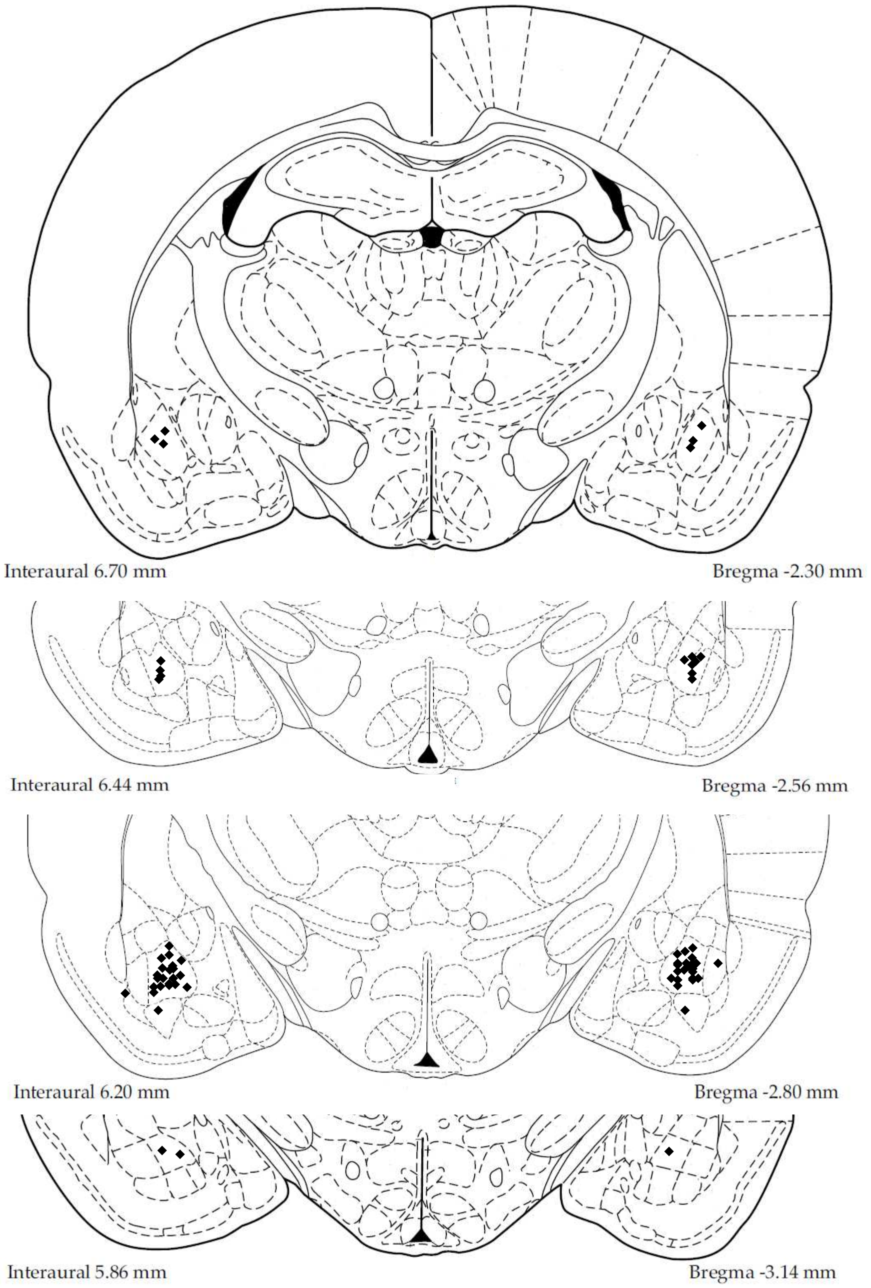


**Supplementary Figure 2:** Location of injection site from rats whose behavioural data were included in the statistical analysis for the PcTx1 experiment. The coordinate is posterior to bregma. Adapted from Paxinos, G. & Watson, C. The Rat Brain in Stereotaxic Coordinates, 7th Edition. New York: Academic Press, 2013.

**Acid-Sensing Ion Channel 1a in the amygdala is involved in pain and anxiety-related behaviours associated with arthritis**

Youssef Aissouni, Abderrahim El Guerrab, Al-Madhy Hamieh, Jeremy Ferrier, Maryse Chalus, Diane Lemaire, Stéphanie Grégoire, Monique Etienne, Alain Eschalier, Denis Ardid, Eric Lingueglia ,Fabien Marchand

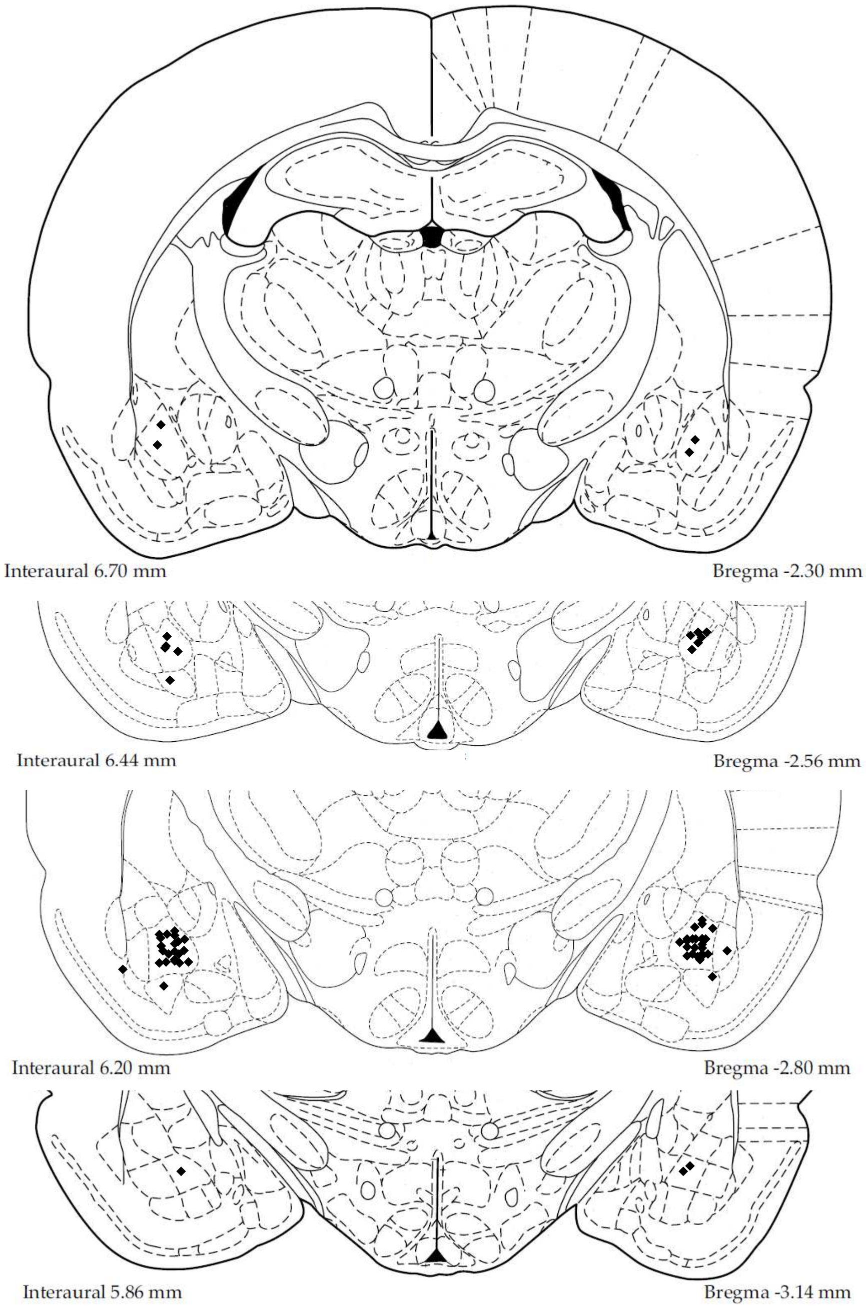


**Supplementary Figure 3:** Location of injection site from rats whose behavioural data were included in the statistical analysis for the Manbalgin-1 experiment. The coordinate is posterior to bregma. Adapted from Paxinos, G. & Watson, C. The Rat Brain in Stereotaxic Coordinates, 7th Edition. New York: Academic Press, 2013.

**Acid-Sensing Ion Channel 1a in the amygdala is involved in pain and anxiety-related behaviours associated with arthritis**

Youssef Aissouni, Abderrahim El Guerrab, Al-Madhy Hamieh, Jeremy Ferrier, Maryse Chalus, Diane Lemaire, Stéphanie Grégoire, Monique Etienne, Alain Eschalier, Denis Ardid, Eric Lingueglia ,Fabien Marchand

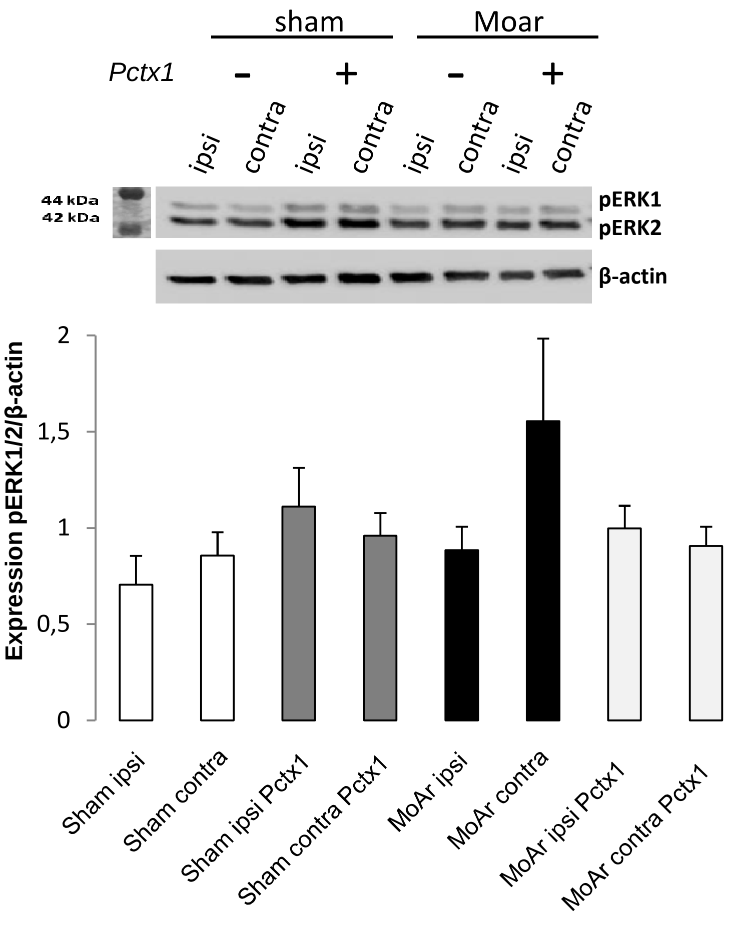


**Supplementary Figure 4:** Expression of phospho-p44/p42 MAPK (ERK1/2) (Cell signaling #4976, 1:1,000) in ipsilateral and contralateral BLA of sham and MoAr rats treated with saline or PcTx1 evaluated by Western blot. Representative Western blot (upper panel) and densitometric quantification of phospho-ERK1/2 of sham and MoAr rats treated with saline or PcTx1 (bottom panel). All phospho bands of p-ERK1/2 were normalized relative to β-actin (Sigma-Aldrich, 1:5,000) and expressed as individual values.
